# Gonadotoxicity of immunotherapy and targeted agents in patients with cancer and impact on subsequent pregnancies

**DOI:** 10.1093/humrep/deaf096

**Published:** 2025-06-07

**Authors:** Luca Arecco, Luciana de Moura Leite, Gabriella Gentile, Kristina Jankovic, Mihaela Stana, Silvia Ottonello, Graziana Scavone, Stefano Spinaci, Matteo Lambertini

**Affiliations:** Department of Internal Medicine and Medical Specialties (DIMI), School of Medicine, University of Genova, Genova, Italy; Université libre de Bruxelles (ULB), Hôpital Universitaire de Bruxelles (H.U.B), Institut Jules Bordet, Academic Trials Promoting Team, Bruxelles, Belgium; Department of Medical Oncology, A.C. Camargo Cancer Center, São Paulo, SP, Brazil; Department of Radiological, Oncological and Pathological Sciences, Sapienza-University of Rome, Rome, Italy; Clinic of Oncology, University Clinical Center Nis, Nis, Serbia; Department of Medical Oncology, Elysee Hospital, Alba Iulia, Alba, Romania; Department of Medical Oncology, U.O.C. Clinica di Oncologia Medica, IRCCS Ospedale Policlinico San Martino, Genova, Italy; Department of Medical Oncology, U.O.C. Clinica di Oncologia Medica, IRCCS Ospedale Policlinico San Martino, Genova, Italy; Department of Breast Surgery, Ospedale Villa Scassi ASL3, Genova, Italy; Department of Internal Medicine and Medical Specialties (DIMI), School of Medicine, University of Genova, Genova, Italy; Department of Medical Oncology, U.O.C. Clinica di Oncologia Medica, IRCCS Ospedale Policlinico San Martino, Genova, Italy

**Keywords:** assisted reproduction, counseling, gonadotoxicity, cancer treatments, young patients, immunotherapy, targeted agents, female infertility, male infertility, ovarian reserve

## Abstract

In recent years, cancer treatment has been revolutionized by the introduction of many novel drugs, including immunotherapy and targeted agents, which have significantly improved the prognosis of patients with different solid tumors. While the role of traditional cytotoxic agents on fertility and reproductive health of patients with cancer is currently well established, the impact of novel treatments remains an unmet medical need and a subject of concern. Limited clinical evidence exists to date on the potential gonadotoxicity of targeted agents and immunotherapy. However, in preclinical male and female animal models, several new treatments have demonstrated the potential to affect reproductive capacity. Hence, the possible impact of these treatments on patients’ reproductive potential should be urgently addressed. This work aims to review the most recent evidence regarding the gonadotoxicity of immunotherapy and novel targeted agents from the mechanisms of action of these treatments to the preclinical and clinical available data, as well as the implications on chances and risks of subsequent pregnancies. The final aim is to provide a useful tool to both physicians and patients for an informed decision-making process regarding fertility preservation and family planning before and after exposure to the new anticancer treatments.

## Graphical Abstract

**Figure:**
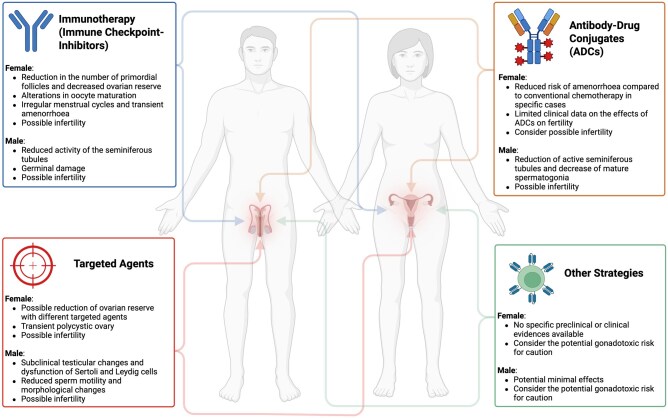
**Classification and main gonadotoxic effect of novel anticancer agents on females and males.** Created in BioRender (https://BioRender.com). The original figure is available in BioRender (https://BioRender.com/w65w494).

## Introduction

Systemic anticancer treatments may negatively impact patients’ reproductive function, potentially leading to gonadal insufficiency and infertility in both women and men (Oktay *et al.*, 2018; ESHRE Guideline Group on Female Fertility Preservation *et al.*, 2020; Lambertini *et al.*, 2020). In women, premature ovarian insufficiency (POI), characterized by early cessation of ovarian function, may result from the direct or indirect treatment-induced damage to ovarian follicles (Chon *et al.*, 2021); similarly, anticancer therapies in male patients may impair gonadal function and spermatogenesis, with subsequent risk of infertility (Dohle, 2010; Perachino *et al.*, 2020).

Immunotherapy and targeted agents, including but not limited to monoclonal antibodies, antibody–drug conjugates (ADCs), tyrosine kinase inhibitors (TKIs), cyclin-dependent kinase 4/6 (CDK4/6) inhibitors, poly ADP-ribose phosphate (PARP) inhibitors, are new effective available anticancer treatments that are characterized by different mechanisms of action than traditional chemotherapy (Mayer *et al.*, 2017; Liu *et al.*, 2024). However, the impact of these new treatments on gonadal function and fertility is very limited and still under investigation (Gentile *et al.*, 2024). Some concerns exist about the reproductive safety of immunotherapy in both men and women (Garutti *et al.*, 2021; Kim *et al.*, 2022); similarly, novel therapies which target specific molecules involved in tumorigenesis and growth may also interfere with reproductive function, particularly if they involve molecular pathways that are also crucial for gonadal regulation (Gentile *et al.*, 2024).

Considering that all these novel treatments are already standard of care and will become widely used in the curative setting, it is of paramount importance to fully understand their impact on fertility, to provide comprehensive information and personalized oncofertility counseling to patients with cancer wishing to preserve fertility and have a subsequent pregnancy after the end of treatment.

## Gonadotoxicity of novel anticancer therapies in male patients

The analysis of semen is essential for the assessment of male fertility. The impact of systemic anticancer therapies on spermatogenesis can be manifested in a variety of conditions, such as azoospermia, oligozoospermia, asthenozoospermia, or teratozoospermia (i.e. increased abnormal sperm count) (Himpe *et al.*, 2023). In general, male gonadotoxicity is expressed as the risk of developing prolonged azoospermia and is classified into three categories according to the risk of prolonged azoospermia (Santaballa *et al.*, 2022). When semen analysis is not available, levels of gonadotropins like FSH, LH, testosterone, and inhibin B can provide an indirect assessment of fertility (Santaballa *et al.*, 2022). Germ cell loss results in a decrease in the secretion of inhibin B by Sertoli cells and, consequently, an increase in FSH levels (Shah *et al.*, 2021; Himpe *et al.*, 2023).

The most commonly performed form of fertility preservation in males is the cryopreservation of sperm prior to the start of anticancer treatments regardless of age, stage of disease, and type of treatment the patient will receive (Brannigan *et al.*, 2021). However, while for traditional gonadotoxic treatments the risk is well established as well as the indication to sperm cryopreservation, uncertainties exist for the new treatments (Brannigan *et al.*, 2021).

### Immunotherapy

Immunotolerance plays a crucial role in spermatogenesis, relying on a delicate balance between the immune-privileged environment (maintained by the blood–testis barrier, testosterone, resident testicular macrophages, and T-lymphocytes), and a robust local innate immune response to protect newly formed germ cells (Nasr *et al.*, 2005; Chen *et al.*, 2016). Immune checkpoint inhibitors (ICIs) antagonize inhibitory receptors and ligands that mediate T-cell activity, like the cytotoxic T-lymphocyte-associated antigen 4 (CTLA-4), programmed cell death protein 1 and its ligand (PD-1/PD-L1), and lymphocyte-activation gene 3 (LAG-3), thus promoting tumor-specific immune response, but also potentially triggering immune-related adverse events (irAEs) (Waldman *et al.*, 2020). Therefore, ICIs could directly or indirectly impact gonadal function by causing primary hypogonadism or affecting the hypothalamic–pituitary–gonadal axis (Garutti *et al.*, 2021; Özdemir, 2021). CTLA-4 and LAG-3 are expressed in human pituitary cells, while their presence in gonadal tissue is less documented (Thudium *et al.*, 2022). On the other hand, PD-1/PD-L1 are poorly detected in pituitary cells and have limited expression in testicular tissue (Fankhauser *et al.*, 2015). The real incidence of ICI-induced male gonadotoxicity is uncertain, as all data come from labeling information in animal models or from case reports and case series (Brunet-Possenti *et al.*, 2017; Quach *et al.*, 2019; Scovell *et al.*, 2020).

A postmortem study revealed a high incidence of impaired spermatogenesis in six out of seven (86%) men treated with anti-PD-1 and/or anti-CTLA-4 agents; however, these results were confounded by the use of other concomitant therapies, including serine/threonine‐protein kinase B‐Raf (BRAF) and MEK inhibitors as well as interleukin 2 (Scovell *et al.*, 2020). On the contrary, a cross-sectional study reported only 1 out 22 (4.5%) cases of azoospermia after exposure to ICIs (Salzmann *et al.*, 2021). Histopathology of the ejaculate showed an inflammatory infiltrate after 1 year of treatment with anti-PD-1 with or without anti-LAG-3 (Salzmann *et al.*, 2021). Two other case reports described ICI-induced orchitis and infertility 2 years after treatment with ICIs (Brunet-Possenti *et al.*, 2017; Quach *et al.*, 2019; Rabinowitz *et al.*, 2021).

Regarding changes in hormone levels, among 49 patients treated with ICIs for melanoma, 34 (69%) displayed low testosterone levels during treatment; however, two-thirds also had low or unknown baseline measures (Peters *et al.*, 2021).

Even though data on hypophysitis were extensively collected from clinical trials (demonstrating an incidence between 0.1% and 10%; Barroso-Sousa *et al.*, 2018; Bai *et al.*, 2020), information on hypogonadotropic hypogonadism derives only from retrospective studies, where it seems to occur in around 76% of patients with pituitary irAEs, of whom only half appear to have chances of recovering (Faje *et al.*, 2014; Albarel *et al.*, 2015; Chang *et al.*, 2019).

**Figure 1.**
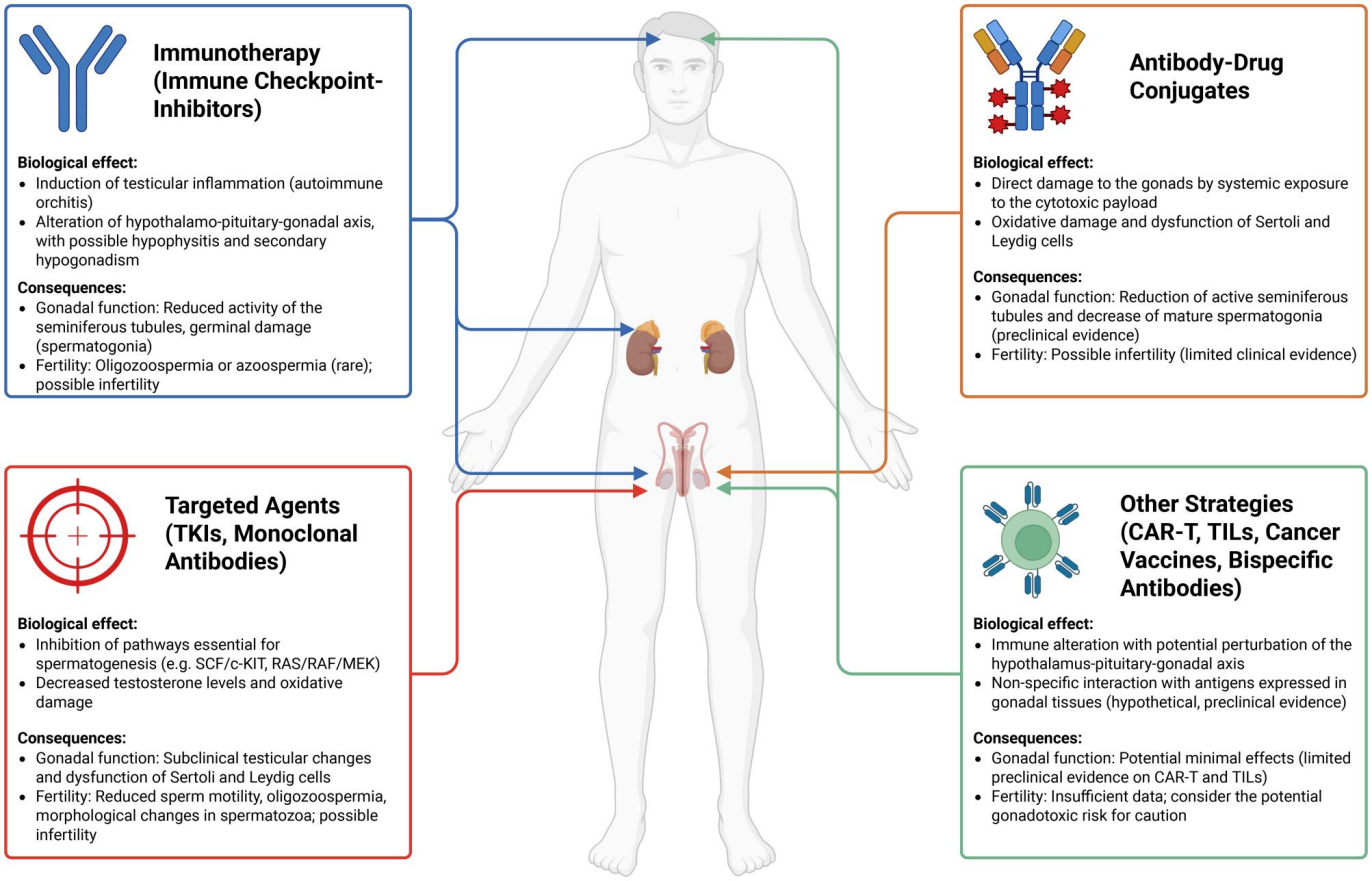
**Biological, gonadal, and fertility consequences of the main targeted agents for oncological treatments in males.** TKIs, tyrosine-kinase inhibitors; SCF/c-KIT, stem cell factor/c-KIT; RAS, rat sarcoma virus; RAF, Rapidly Accelerated Fibrosarcoma; MEK, mitogen-activated protein kinase; CAR-T, Chimeric antigen receptor-T; TILs, tumor-infiltrating lymphocytes. Created in BioRender (https://BioRender.com). The original figure is available in BioRender (https://BioRender.com/w65w494).

In conclusion, although evidence to date is scarce and of low quality, it appears that ICIs may have a negative impact in men both directly on gonadal health and indirectly through alterations in the hypothalamic–pituitary axis. Therefore, comprehensive oncofertility counseling is recommended prior to initiating treatment with ICIs in male patients, regardless of their combination with other gonadotoxic agents (Fig. 1).

### Targeted agents

Targeted agents, including small molecules and monoclonal antibodies, inhibit tumor growth by interacting with specific cancer cell antigens, receptors, or signaling pathways. Despite their selectivity, they can still impact gonadal function and fertility (Liu *et al.*, 2024). In males, these drugs may disrupt spermatogenesis by interfering with essential signaling pathways, increasing oxidative stress, and impairing Sertoli and Leydig cell function, affecting testicular integrity and testosterone production (Shah *et al.*, 2021). For instance, stem cell factor (SCF)/c-KIT is known to communicate with downstream signaling targets RAS/RAF/MEK/ERK, thus regulating testosterone synthesis, germ cell meiosis, sperm migration, and maturation, the latter also facilitated by platelet-derived growth factor receptor (PDGFR) (Gnessi *et al.*, 1995; Mauduit *et al.*, 1999; Basciani *et al.*, 2002). Other possible targets evaluated in preclinical models are insulin-like growth factor 1 receptor, linked to Sertoli cell proliferation (Cannarella *et al.*, 2018), MNNG HOS transforming gene (MET), epidermal growth factor receptor (EGFR), vascular endothelial growth factor (VEGF), and anaplastic lymphoma kinase, with different roles in gonadal steroidogenesis (Evaul and Hammes, 2008; Shiraishi and Ascoli, 2008; Witek *et al.*, 2015; Vita *et al.*, 2024), proto-oncogene tyrosine-protein kinase ROS (ROS1) and rearranged during transfection proto-oncogene (RET) involved in epididymis differentiation (Jun *et al.*, 2014) and in spermatogonia stem cells self-renewal (Oatley *et al.*, 2007), respectively. Notably, tyrosine phosphorylation, inhibited by multiple inhibitors (TKIs), is vital to spermatozoid capacitation (Krapf *et al.*, 2010; Kwon *et al.*, 2019); however, clinical findings are limited to older TKIs only. Imatinib (BCL-Abl inhibitor) has conflicting effects on spermiogenesis and hypogonadism, but without apparent adverse fertility outcomes (Seshadri *et al.*, 2004; Chang *et al.*, 2017). Sunitinib and pazopanib were linked to reduced testosterone levels (Bastin *et al.*, 2019; Volta *et al.*, 2023). Crizotinib appears to be associated with reversible hypogonadism in a high proportion of patients (Sargis and Salgia, 2015). Gefitinib use was reported to induce abnormal sperm parameters (Nagamitsu *et al.*, 2021), while vemurafenib seems to have no impact on fertility parameters (Ghezzi *et al.*, 2020); cases of fatherhood without complications during dabrafenib and trametinib therapy for metastatic melanoma have been reported (Cocorocchio *et al.*, 2018).

Second-generation TKIs may not cross the blood–testis barrier and thus they may have reduced effects on male fertility or testosterone production (Rambhatla *et al.*, 2021), but clinical data are lacking. A summary of the available evidence of TKI-induced gonadotoxicity in male patients is reported in Table 1.

**Table 1.** Novel targeted agents and male fertility.

Drug	Targets	Labeling information	Clinical evidence
Dabrafenib Trametinib	BRAF MEK1/2	Dabrafenib: Testicular degeneration Trametinib: No effects reported	2 cases: 1 reduced sperm motility/count (Ghezzi *et al.*, 2020) 1 normal sperm and fatherhood (Cocorocchio *et al.*, 2018)
Encorafenib Binimetinib	BRAF CRAF MEK1/2	Encorafenib: Reduced testis, oligospermia Binimetinib: No effects reported	–
Cabozantinib	VEGFR KIT TRK FLT3 AXL RET MET TIE-2	Hypospermia Decreased reproductive organ weights	–
Vandetanib	EGFR VEGFR RET BRK TIE-2 EPH Src-kinase	Increased testosterone Decreased inhibin B and FSH	Decreased live embryos Increased preimplantation loss in females mated to treated males (Thornton *et al.*, 2012)
Lenvatinib	VEGFR FGFR PDGFR KIT RET	Testicular hypocellularity	–
Selpercatinib	RET VEGFR	Testicular degeneration Reduced sperm motility No impact on mating and/or fertility	–
Vorasidenib	IDH1/2	Testicular atrophy Prostate and seminal vesical atrophy	–
Belzutifan	HIF-2α	Testicular degeneration/atrophy Reduced fertility	–
Osimertinib	EGFR HER2-4 ACK1 BLK	Testis degeneration Pre-implantation loss in females mated to treated males	–
Alectinib	ALK RET	No effects reported	11/27 (41%) patients with low testosterone and most patients with symptomatic hypogonadism, not compensated by LH and FSH (Vita *et al.*, 2024)
Lorlatinib	ALK ROS1 TYK1 FPS TRK FAK ACK	Reversible lower testicular, epididymal, prostate weights Testicular degeneration/atrophy Prostatic atrophy Epididymal inflammation	–
Entrectinib	ALK ROS1 Pan-TRK	Decreased prostate weight	–
Larotrectinib	Pan-TRK	No effects reported	–

ACK1, activated Cdc42-associated kinase 1; ALK, anaplastic lymphoma kinase; AXL, AXL receptor tyrosine kinase; BLK, B lymphocyte kinase; BRAF, serine/threonine-protein kinase B-Raf; BRK, breast tumor kinase; CRAF (or c-CRAF), Raf-1 proto-oncogene; EGFR, epidermal growth factor receptor; EPH, ephrin type receptor; FAK, focal adhesion kinase; FPS, proto-oncogene c-Fes/Fps; FGFR, fibroblast growth factor receptor; FLT3, Fms-like tyrosine kinase 3; HIF-2α, hypoxia-inducible factor 2 alpha; HER2-4, human epidermal growth factor receptors 2–4; IDH1/2, isocitrate dehydrogenases 1 and 2; KIT, KIT proto-oncogene; MEK1/2, mitogen-activated protein kinase kinases 1 and 2; MET, hepatocyte growth factor receptor (c-Met); PDGFR, platelet-derived growth factor receptor; RET, rearranged during transfection proto-oncogene; ROS1, proto-oncogene tyrosine-protein kinase ROS; SRC, non-receptor tyrosine kinase SRC; TIE-2, angiopoietin-1 receptor; TRK, tropomyosin receptor tyrosine kinase; TYK1, leukocyte receptor tyrosine kinase 1; VEGFR, vascular endothelial growth factor receptor.

In conclusion, several mechanisms of action of the targeted agents may lead to gonadal damage through direct and indirect effects on sperm production; therefore, while waiting for more clinical data, these treatments should be considered potentially gonadotoxic, and oncofertility counseling is recommended prior to treatment initiation (Fig. 1).

### Antibody-drug conjugates

ADCs are novel anticancer therapies that combine a humanized antibody directed against a specific antigen linked with a potent cytotoxic drug (Chau *et al.*, 2019). The antibody specifically binds to antigens on the surface of cancer cells, allowing the drug to be delivered directly to the tumor, minimizing damage to healthy cells and enhancing the efficacy of the treatment (Chau *et al.*, 2019; Tarantino *et al.*, 2023).

Although the design of ADCs aims to limit toxic drug exposure only to tumor cells, systemic distribution has been demonstrated, potentially also affecting gonadal cells (Tarantino *et al.*, 2023).

The first ADC approved by the USA Food and Drug Administration (FDA) in solid tumors was trastuzumab emtansine (T-DM1) in breast cancer (Wedam *et al.*, 2020). Since then, there has been a substantial expansion in this drug class with six ADCs approved by the FDA for the treatment of solid tumors: T-DM1 (Wedam *et al.*, 2020), enfortumab vedotin (EV) (Maguire *et al.*, 2024), trastuzumab deruxtecan (T-DXd) (Narayan *et al.*, 2021), sacituzumab govitecan (SG) (Wahby *et al.*, 2021), tisotumab vedotin (TV) (U.S. Food and Drug Administration, 2021), and mirvetuximab soravtansine (Dilawari *et al.*, 2023).

Regarding specifically the treatment of males, the currently approved ADCs are EV, T-DXd, and SG. Despite their growing importance and clinical use, the impact of ADCs on males’ fertility is largely unknown (Lambertini *et al.*, 2020; Gentile *et al.*, 2024).

EV is an ADC indicated for treatment of locally advanced or metastatic urothelial cancer following the phase III trial EV-301 (Powles *et al.*, 2021). In this study, 233 males out of 608 patients enrolled were included but effects on male gonadal toxicity were not reported (Powles *et al.*, 2021). However, in repeat-dose toxicology studies conducted in rats (at exposures similar to those recommended in humans), a decrease in testis and epididymis weights, seminiferous tubule degeneration, spermatid/spermatocyte depletion, and hypospermia/abnormal spermatids in the epididymis were observed. Moreover, the findings in the testis and epididymis did not reverse by the end of the treatment period (Maguire *et al.*, 2024).

T-DXd received approval in male patients for the treatment of HER2-positive advanced gastric or gastroesophageal junction cancer (phase II DESTINY-Gastric01 trial; Shitara *et al.*, 2020) and pretreated metastatic HER2-mutant non-small cell lung cancer (NSCLC) (phase II DESTINY-Lung01 trial (Li *et al.*, 2022), phase II DESTINY-Lung02 trial (Goto *et al.*, 2023)). In the DESTINY-Gastric01 trial, 142 out of 187 patients were males (Shitara *et al.*, 2020), while in the DESTINY-Lung trials, 84 male patients were included. No results regarding male gonadal toxicity are available in these trials (Li *et al.*, 2022; Goto *et al.*, 2023). T-DXd has also recently received approval for the treatment of adult patients with any advanced HER2-positive solid tumors based on results of DESTINY-PanTumor02 (Meric-Bernstam *et al.*, 2024), DESTINY-Lung01 (Li *et al.*, 2022), and DESTINY-CRC02 (Raghav *et al.*, 2024) trials. A total of 192 patients were included in these studies but no data on male gonadotoxicity were reported. However, the FDA warns that T-DXd may impair male fertility in preclinical models: in monkeys, intravenous administration of T-DXd resulted in decreased numbers of round spermatids in the testis (Narayan *et al.*, 2021).

SG received approval for the treatment of advanced urothelial carcinoma following the results of the TROPHY-U-01 trial (Tagawa *et al.*, 2021). No preclinical nor clinical data are available to date concerning male gonadotoxicity of SG.

In conclusion, to date, three different ADCs are approved for the treatment of different solid tumors in male patients, but no clinical data are currently available on their gonadotoxicity. However, based on the characteristics of the drugs (particularly the presence of the cytotoxic payload) and on preclinical data, gonadotoxicity of ADCs cannot be excluded and should be discussed prior to the initiation of treatment (Fig. 1).

### Other strategies

Bispecific antibodies (BsAbs) represent a novel category of anticancer drugs, designed to simultaneously bind to two different antigens, enabling direct recruitment of immune cells against tumor cells or simultaneously blocking two signaling pathways critical for tumor growth (Labrijn *et al.*, 2019).

Tebentafusp, was the first approved BsAb in the treatment of solid tumors (U.S. Food and Drug Administration, 2022). In 121 patients treated with tebentafusp in the pivotal trial, no data on gonadotoxicity were reported (Nathan *et al.*, 2021). The other FDA-approved BsAb for the treatment of solid tumors in male patients affected by advanced NSCLC is amivantamab (Chon *et al.*, 2023), after the results of the phase II PAPILLON (Zhou *et al.*, 2023) and the phase III MARIPOSA-2 (Passaro *et al.*, 2024) trials. Overall, 381 male patients were randomized in these trials, but no results on male gonadotoxicity were reported. In terms of preclinical data, for amivantamab no specific studies have been performed to evaluate the potential gonadotoxicity; however, toxicology studies in monkeys showed no notable effects in the male reproductive organs (Chon *et al.*, 2023). No clinical data are available for tebentafusp (U.S. Food and Drug Administration, 2022).

The mechanism of action of cancer vaccines is based on activating the body’s immune response to fight against tumor cells using specific antigens which are found in the tumor microenvironment (Fan *et al.*, 2023). No data regarding their potential gonadal toxicity in male patients have been reported to date.

Another strategy currently expanding to the treatment of solid tumors is represented by cellular therapies. Treatment with chimeric antigen receptor-T (CAR-T) is not yet approved in solid tumors (Guzman *et al.*, 2023). Tumor-infiltrating lymphocytes (TILs) therapy uses patient’s own T-cells originated from the tumor as an anticancer treatment. FDA approved Lifileucel (LN-144)—the first TILs-therapy—in 2024 for the treatment of advanced melanoma; however, no data regarding male gonadal toxicity were reported (Chesney *et al.*, 2022).

In conclusion, several new strategies are rapidly becoming available in the clinical setting for the treatment of solid tumors in male patients; however, no clinical data are available to date on their impact on male gonadal function and fertility (Fig. 1).

## Gonadotoxicity of novel anticancer therapies in female patients

Systemic anticancer therapies can impact ovarian function through different mechanisms, leading to diminished ovarian reserve, impaired folliculogenesis, and POI. These effects are largely dependent on the type of treatment, cumulative dose, and the patient’s age at the time of exposure. Female gonadotoxicity is often measured by the risk of developing amenorrhea or reduced ovarian reserve, assessed using biomarkers like anti-Müllerian hormone (AMH) or antral follicle count (ESHRE Guideline Group on Female Fertility Preservation *et al.*, 2020; Lambertini *et al.*, 2020).

When direct markers of ovarian reserve are not available, surrogate indicators such as menstrual history and gonadotropin levels can provide an indirect assessment, with loss of ovarian function typically resulting in reduced secretion of estrogens that leads to increased FSH levels and irregular or absent menstrual cycles (ESHRE Guideline Group on Female Fertility Preservation *et al.*, 2020; Lambertini *et al.*, 2020). However, while the gonadotoxic risks of traditional chemotherapy agents are well-documented, uncertainties persist regarding newer treatments such as TKIs, ADCs, and ICIs that may also have distinct direct and indirect gonadal effects (Lambertini *et al.*, 2022; Gentile *et al.*, 2024).

AMH, secreted by granulosa cells, primarily originates from preantral and small antral follicles, making it a valuable quantitative marker of ovarian reserve (Anderson *et al.*, 2022). However, while a higher residual ovarian reserve is associated with improved ART outcomes, it does not directly correlate with natural fertility or embryo viability. Although there may be a relationship between pre-treatment AMH levels and subsequent ovarian reserve after gonadotoxic therapy, its predictive value for post-treatment reproductive capacity remains to be fully determined. The use of FSH has decreased over time due to its limited accuracy as an indirect marker of ovarian reserve and its high intercycle variability (ESHRE Guideline Group on Female Fertility Preservation *et al.*, 2020; Anderson *et al.*, 2022; Santaballa *et al.*, 2022). The incidence of amenorrhea is often used as a surrogate marker for gonadal damage in clinical trials. Gonadotoxicity is generally categorized by the risk of developing post-treatment amenorrhea, with three risk levels: high (>80%), intermediate (20–80%), and low/very low (<20%) (Perachino *et al.*, 2020). However, amenorrhea has significant limitations and should be considered a very poor predictor of fertility: resumption of menses does not guarantee restored fertility as ovarian damage can persist even when menstrual cycles do return or do not temporarily disappear during treatments (Zhao *et al.*, 2014). Additionally, patients may still face early menopause despite temporary recovery of menstrual cycles following treatment completion, and post-treatment fertility can be significantly reduced even with resumption of menses (Lambertini *et al.*, 2020). Therefore, markers like AMH are preferable for a more accurate assessment of gonadotoxicity, as they directly reflect ovarian reserve (Ruddy *et al.*, 2021a).

While the gonadotoxicity of chemotherapy treatments is well established and the risk quantifiable (Lambertini *et al.*, 2020; Martelli *et al.*, 2021), for all new anticancer therapies this risk is much less well known making patients’ counseling highly challenging (Oktay *et al.*, 2018; ESHRE Guideline Group on Female Fertility Preservation *et al.*, 2020; Lambertini *et al.*, 2020).

### Immunotherapy

Immunotherapy, and in particular ICIs, have led to a revolution in the treatment of solid tumors among female patients (Guha *et al.*, 2022), but their impact on the ovaries is debated.

In preclinical models of young adult female mice treated with PD-L1 or CTLA-4 inhibitors, a reduction in ovarian follicular reserve has been demonstrated, with subsequent negative effects on ovulation and oocyte maturation (Winship *et al.*, 2022). Furthermore, injection of anti-PD-1 antibody in immunocompetent mice dramatically reduced the number of primordial follicles with increased CD3^+^ T-cell infiltration, suggesting a pro-inflammatory effect (Xu *et al.*, 2021).

Clinical data on the effects of ICIs on the female reproductive system are limited. When examining the cellular makeup of the female reproductive tract, up to 20% of the cells are immune cells, with 20–40% of CD4^+^ and CD8^+^ T lymphocytes expressing PD-L1, with variations based on anatomical location and menopausal status (Monin *et al.*, 2020). IrAEs affect ∼10% of patients receiving ICIs, with premenopausal women being more likely to develop endocrinological irAEs than postmenopausal women (Duma *et al.*, 2019; Li *et al.*, 2024). Hypothyroidism, the most common endocrinological irAEs (affecting 15% of patients treated with ICIs) impairs menstrual cycle and ovulation (Koyyada and Orsu, 2020). Hypophysitis, despite being less frequent, affects the secretion of FSH and LH, crucial for a normal menstrual cycle and for achieving pregnancy (Chang *et al.*, 2019). The ECOG-ACRIN E1609 trial examined ovarian reserve in women aged 20–35 years treated with 3 or 10 mg/kg ipilimumab for melanoma. Results showed significant decreases in AMH, estrogen, and LH levels after 8 months (Buchbinder *et al.*, 2023).

**Figure 2.**
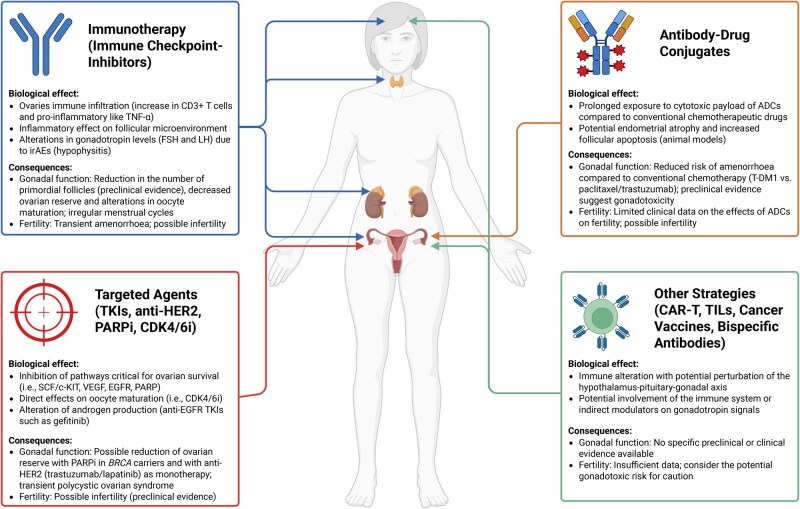
**Biological, gonadal, and fertility consequences of the main targeted agents for oncological treatments in females.** TNF-α, tumor necrosis factor alpha; FSH, follicle-stimulating hormone; LH, luteinizing hormone; irAEs, immune-related adverse events; TKIs, tyrosine-kinase inhibitors; HER2, human epidermal growth factor receptor 2; PARPi, poly ADP-ribose phosphate inhibitors; CDK4/6i, cyclin-dependent kinase 4/6 inhibitors; SCF/c-KIT, stem cell factor/c-KIT; VEGF, vascular endothelial growth factor; EGFR, epidermal growth factor receptor; BRCA, breast cancer gene; ADCs, antibody-drug conjugates; T-DM1, trastuzumab emtansine; CAR-T, Chimeric antigen receptor-T; TILs, tumor-infiltrating lymphocytes. Created in BioRender (https://BioRender.com). The original figure is available in BioRender (https://BioRender.com/w65w494).

In conclusion, with the currently available data, it is not possible to exclude long-term impact of immunotherapy agents on ovarian reserve. Therefore, this limited evidence and concern should be discussed with all premenopausal patients before starting any ICI, alone or in combination with other gonadotoxic treatments (Fig. 2).

### Targeted agents

The impact of most of the novel targeted agents on female fertility is unknown despite their effects on the molecular pathways involved in gonadal development and maturation. Among them, (SCF)/c-KIT with downstream RAS/RAF/MEK/ERK signaling pathway enables survival of the ovaries during early folliculogenesis and cell proliferation (Carlsson *et al.*, 2006), PDGF supports follicular development (Nilsson *et al.*, 2006), EGFR and RAF regulate oocyte maturation (Muslin *et al.*, 1993; Schneider and Wolf, 2008), VEGF controls oocyte migration (Holmes and Zachary, 2005), while PARP genes protect oocytes against DNA damage (Turan and Oktay, 2020).

Considering the crucial role of these targets in the development and health of female gonads, many of the targeted agents developed in the last years have shown a potential gonadotoxicity in animals through their inhibition of these targets (Rosario *et al.*, 2022; Gentile *et al.*, 2024).

PARP inhibition with olaparib depletes the primordial follicle oocyte pool by 36% in mouse models (Winship *et al.*, 2020). Young *BRCA* carriers seem to have a reduced ovarian reserve already before anticancer treatments (Turan and Oktay, 2020); however, these data, and particularly those on the potential additional treatment-induced gonadotoxicity, remain limited and controversial to date.

The limited clinical data in patients with breast cancer treated with trastuzumab and/or lapatinib suggest that anti-HER2 agents appear to be safe on the ovaries (Ruddy *et al.*, 2015; Lambertini *et al.*, 2019), and they seem to only slightly reduce ovarian reserve when administered as single agent without concurrent chemotherapy (Lambertini *et al.*, 2023).

CDK4/6 inhibitors may play an influential role in regulating meiotic progression in mouse oocytes (Scavone *et al.*, 2023). In terms of clinical data, the CDK4/6 inhibitor palbociclib did not appear to significantly affect estradiol and FSH levels in patients affected by breast cancer (Furlanetto *et al.*, 2022), while no data are available with the two approved CDK4/6 inhibitors, abemaciclib and ribociclib, in the adjuvant setting, (Scavone *et al.*, 2023).

A few studies have described a transient ovarian failure in patients treated with bevacizumab (anti-VEGF antibody) (Imai *et al.*, 2017) and decreased androgen levels associated with the anti-EGFR TKI, gefitinib (Nishio *et al.*, 2005). Ovarian dysfunction has been reported in cases with pazopanib (De Sanctis *et al.*, 2019), imatinib (Christopoulos *et al.*, 2008), and lenvatinib (Aoki *et al.*, 2023).

Despite these conflicting data, individual cases of pregnancy during therapy with different TKIs have also been described (Su *et al.*, 2016; Thomas *et al.*, 2018; Soberanis Pina *et al.*, 2023; Kato *et al.*, 2024). Therefore, the real impact of the new molecules on fertility and chances of pregnancy in young patients to date remains unclear and new studies are awaited to properly address this issue. A summary of data on ovarian toxicity in women treated with novel targeted agents is shown in Table 2 and Fig. 2.

**Table 2.** Novel targeted agents and female fertility.

Drug	Targets	Labeling information	Clinical evidence
Pertuzumab	HER2	No effects reported	–
Neratinib	EGFR HER2 HER4	No effects reported	–
Ribociclib	CDK4/6	No effects reported	–
Abemaciclib	CDK4/6	No effects reported	–
Vemurafenib	BRAF	No effects reported	–
Dabrafenib	BRAF	Decreased corpus luteum Fertility reduction	–
Trametinib	MEK1/2	Increased follicular cysts Decreased corpus luteum	–
Cobimetinib	MEK1/2	Increased apoptosis/necrosis of corpus luteum Increased apoptosis/necrosis of vaginal epithelial cells	–
Cabozantinib	VEGFR KIT TRK FLT3 AXL RET MET TIE-2	Absence of corpus luteum Ovarian necrosis Reduced fertility	–
Vandetanib	EGFR VEGFR RET BRK TIE-2 EPH Src-kinase	Decrease number of corpus luteum Menstrual cycle irregularity Pregnancy rate decrees Increased implantation loss	Case: Pregnancy under vandetanib (Thomas *et al.*, 2018)
Lenvatinib	VEGFR FGFR PDGFR KIT RET	Follicular atresia Decreased incidence of menses	Case: Premature ovarian insufficiency (Aoki *et al.*, 2023)
Selpercatinib	RET VEGFR	Decreased number of menstrual cycles Decreased/absent corpus luteum	–
Vorasidenib	IDH1/2	Uterus and vaginal atrophy Decreased/absent corpus luteum Increased atretic follicles Menstrual cycle alterations	–
Belzutifan	HIF-2α	No effects reported	–
Osimertinib	EGFR HER2-4 ACK1 BLK	Degeneration corpus luteum Uterus and vaginal epithelial thinning	Case: Pregnancy under osimertinib (Soberanis Pina *et al.*, 2023)
Alectinib	ALK RET	No effects reported	Case: Pregnancy under alectinib (Kato *et al.*, 2024)
Lorlatinib	ALK ROS1 TYK1 FPS TRK FAK ACK	No data reported	Case: Pregnancy under lorlatinib (Mawet *et al.*, 2023)
Entrectinib	ALK ROS1 Pan-TRK	No effects reported	–
Larotrectinib	Pan-TRK	Decreased fertility Decreased corpus luteum Decreased uterine weight Increased anestrus	–
Erlotinib	EGFR	No effects reported	–
Cetuximab	EGFR	Irregular/absent menstrual cycles	–
Panitumumab	EGFR	Prolonged menstrual cycles Amenorrhea Delay in progesterone peak	–
Crizotinib	ALK HGFR c-Met ROS1 RON	Necrosis of ovarian follicles	Case: Pregnancy under crizotinib and ovarian stimulation (Su *et al.*, 2016)
Sunitinib	PDGFR VEGFR KIT FLT3 CSF-1R RET	Decreased follicular development Endometrial atrophy (monkey) No fertility effect (rats)	–
Sorafenib	c-CRAF BRAF KIT FLT-3 RET RET/PTC VEGFR-1 PDGFR-ß	Necrosis of corpus luteum Arrested follicular development	–

ACK1, activated Cdc42-associated kinase 1; ALK, anaplastic lymphoma kinase; AXL, AXL receptor tyrosine kinase; BLK, B lymphocyte kinase; BRAF, serine/threonine-protein kinase B-Raf; BRK, breast tumor kinase; CDK4/6, cyclin-dependent kinases 4 and 6; CRAF (or c-CRAF), Raf-1 proto-oncogene; CSF-1R, colony stimulating factor 1 receptor; EGFR, epidermal growth factor receptor; EPH, Ephrin type receptor; FAK, focal adhesion kinase; FPS, proto-oncogene c-Fes/Fps; FGFR, fibroblast growth factor receptor; FLT-3 (or FLT3), Fms-like tyrosine kinase 3; HGFR (also MET or c-Met), hepatocyte growth factor receptor; HIF-2α, hypoxia-inducible factor 2 alpha; HER2, human epidermal growth factor receptor 2; HER4, human epidermal growth factor receptor 4; HER2-4, human epidermal growth factor receptors 2–4; HER, human epidermal growth factor receptor; IDH1/2, isocitrate dehydrogenases 1 and 2; KIT, KIT proto-oncogene; MEK1/2, mitogen-activated protein kinase kinases 1 and 2; PDGFR, platelet-derived growth factor receptor; PDGFR-β, platelet-derived growth factor receptor beta; PTC, papillary thyroid carcinoma; RET, rearranged during transfection proto-oncogene; RET/PTC, RET rearrangement/fusion commonly found in papillary thyroid carcinoma; RON, Receptor Nantais Origin; ROS1, proto-oncogene tyrosine-protein kinase ROS; SRC, non-receptor tyrosine kinase SRC; TIE-2, angiopoietin-1 receptor; TRK, tropomyosin receptor tyrosine kinase; Pan-TRK, A term referring collectively to TRKA, TRKB, and TRKC; TYK1, Leukocyte receptor tyrosine kinase 1; VEGFR, vascular endothelial growth factor receptor; VEGFR-1, vascular endothelial growth factor receptor 1.

### Antibody–drug conjugates

ADCs are a growing category of drugs employed in the treatment of solid tumors in women. ADCs currently approved for use in female subjects include T-DM1 (Wedam *et al.*, 2020), EV (Maguire *et al.*, 2024), T-DXd (Narayan *et al.*, 2021), SG (Wahby *et al.*, 2021), TV, and mirvetuximab soravtansine (Dilawari *et al.*, 2023).

Adjuvant T-DM1 has been approved based on the phase III KATHERINE trial (Wedam *et al.*, 2020). While this study did not report data on gonadotoxicity, some information on the gonadotoxic impact of T-DM1 was reported in the ATEMPT trial (Ruddy *et al.*, 2021b). In this study, data on chemotherapy-related amenorrhea (CRA) were reported in 76 out of the 123 premenopausal women enrolled. At 18 months after treatment completion, CRA was observed in 24% of patients in the T-DM1 arm, compared with 50% in the paclitaxel-trastuzumab arm (*P* = 0.045). However, a progressive increase in CRA rate was also observed in patients treated with T-DM1, and can be attributed to the duration of cytotoxic therapy (1 year of treatment in T-DM1 arm vs 12 weeks in the paclitaxel and trastuzumab arm) (Ruddy *et al.*, 2021b).

T-DXd and SG are two more recently approved ADCs for the treatment of different solid tumors, including advanced breast cancer (Narayan *et al.*, 2021; Wahby *et al.*, 2021), advanced gastric, or gastroesophageal junction cancer (Shitara *et al.*, 2020), pretreated metastatic HER2-mutant NSCLC (Li *et al.*, 2022), or advanced urothelial cancer (Tagawa *et al.*, 2021). No specific data about T-DXd or SG for premenopausal women are available from the pivotal trials, nor have dedicated fertility studies been conducted. However, preclinical data showed that, in monkeys, SG can cause adverse effects on female reproductive organs, including endometrial atrophy, uterine bleeding, increased follicular ovarian atresia, and vaginal epithelial cell atrophy (Wahby *et al.*, 2021).

EV is approved for the treatment of advanced urothelial carcinoma (Maguire *et al.*, 2024); preclinical data indicated a potential impairment of female reproductive function and fertility in mice (Maguire *et al.*, 2024). TV, approved for the treatment of recurrent or metastatic cervical cancer (U.S. Food and Drug Administration, 2021), was associated with adverse ovarian effects when administered to sexually immature monkeys, including a decrease or absence of secondary and tertiary ovarian follicles; these effects showed a trend toward recovery after the end of treatment. No fertility preclinical or clinical studies have been performed to date for mirvetuximab soravtansine, an ADC approved for the treatment of epithelial ovarian, fallopian tube, or primary peritoneal cancer (Dilawari *et al.*, 2023).

In conclusion, limited data exist on T-DM1 gonadotoxicity in women, while no clinical data for other ADCs are available. Given their mechanism and preclinical findings, oncofertility counseling is recommended before treatment. Assessing gonadotoxicity is crucial, especially with ongoing early-stage trials, notably for T-DXd (Geyer *et al.*, 2022; Harbeck *et al.*, 2024) (Fig. 2).

### Other strategies

The only FDA-approved BsAbs for the treatment of solid tumors in female patients are amivantamab and tebentafusp. Overall, 209 females received BsAbs in the two pivotal trials, but no results on female gonadotoxicity were reported (Nathan *et al.*, 2021; Zhou *et al.*, 2023). However, in repeat-dose toxicology studies in monkeys, amivantamab showed no notable effects on female reproductive organs (Chon *et al.*, 2023). No preclinical or clinical studies have been conducted on the impact of tebentafusp on female gonads to date (U.S. Food and Drug Administration, 2022).

Regarding cancer vaccines, since these novel drugs aim to enhance the immune response rather than directly target cancer cells, they are less likely to cause gonadotoxicity; however, the induced immune response could directly or indirectly affect ovarian function. Nevertheless, so far, no clinical data on these agents are available.

Other strategies, like chimeric antigen receptor-T (CAR-T) cells (Guzman *et al.*, 2023) or TILs therapy (Chesney *et al.*, 2022), have not reported clinical data on female gonadal toxicity.

In conclusion, further studies are needed to assess the impact of BsAbs, cancer vaccines, and cell therapies on female fertility. Their gonadotoxic risk remains uncertain but cannot be excluded (Fig. 2).

## Fertility preservation and post-treatment pregnancies in patients exposed to novel anticancer therapies

Major international guidelines recommend performing oncofertility counseling as early as possible after diagnosis in all patients, irrespective of the type and stage of cancer (Oktay *et al.*, 2018; ESHRE Guideline Group on Female Fertility Preservation *et al.*, 2020; Lambertini *et al.*, 2020). Fertility preservation techniques can ensure, in both men and women, a higher likelihood of subsequent pregnancy following gonadotoxic treatments (Oktay *et al.*, 2018; ESHRE Guideline Group on Female Fertility Preservation *et al.*, 2020; Lambertini *et al.*, 2020). However, with the constant introduction of novel anticancer drugs, oncofertility counseling in patients with solid tumors has reached a new dimension (Gentile *et al.*, 2024). These treatments, although extremely effective, may have a variable impact on fertility in the medium- and long-term, and while for traditional chemotherapy the gonadotoxic risk is established, for all new treatments, the evidence to date are insufficient to make clear recommendations (Massarotti *et al.*, 2019).

Sperm cryopreservation is the standard fertility preservation method for male patients before treatment. It is quick, widely used, and effective, even before therapies with uncertain gonadal effects. It should be highlighted that men with systemic diseases (including cancer) often show poor baseline sperm parameters (Kasman *et al.*, 2020); however, this should not discourage sperm cryopreservation before treatment start, since IVF requires only a single sperm per oocyte, and ICSI needs only a few sperm to achieve successful fertilization (Oktay *et al.*, 2018; Lambertini *et al.*, 2020).

In female patients, cryopreservation of oocytes or embryos is the first option for fertility preservation that should be discussed. Before starting treatments, female patients can undergo ovarian stimulation to collect oocytes or embryos, which can be cryopreserved for future use. This procedure is safe (Arecco *et al.*, 2022) and effective (Ní Dhonnabháin *et al.*, 2022) and must be the first choice in post-pubertal patients who can delay treatment start by 2–3 weeks (ESHRE Guideline Group on Female Fertility Preservation *et al.*, 2020; Lambertini *et al.*, 2020; Arecco *et al.*, 2022). Cryopreservation of ovarian tissue involves removing and freezing ovarian tissue fragments before starting treatments. Subsequently, the tissue can be reimplanted to restore ovarian function and fertility. It is preferably reserved for young patients (≤35 years of age) with normal ovarian reserve and contraindications to oocyte/embryo preservation, including those with urgent need to start anticancer therapies (ESHRE Guideline Group on Female Fertility Preservation *et al.*, 2020; Lambertini *et al.*, 2020; Ní Dhonnabháin *et al.*, 2022).

Ovarian function suppression with the use of GnRH agonist during chemotherapy has been shown to protect ovarian function during classical cytotoxic treatments; it should not be considered a fertility preservation strategy *per se* and, thus, it should not replace cryopreservation options (Lambertini *et al.*, 2018, 2020; ESHRE Guideline Group on Female Fertility Preservation *et al.*, 2020). The efficacy and need of this strategy during treatment with new therapies are currently unknown.

The chances of pregnancy after anticancer therapies depend on several factors, including the type and duration of treatment, the patient’s age, and residual fertility (ESHRE Guideline Group on Female Fertility Preservation *et al.*, 2020; Lambertini *et al.*, 2020). Many modern anticancer treatments are teratogenic and contraindicated during pregnancy. The washout period from completion of systemic anticancer therapies to have a safe pregnancy is variable depending on the class of agents (Perachino *et al.*, 2022; Arecco and Lambertini, 2023; Arecco *et al.*, 2023). Data suggest no increased risk of congenital malformations after chemotherapy, but there may be higher rates of low birth weight, preterm birth, and small-for-gestational-age infants, especially if conception occurs within a year from treatment completion (Hartnett *et al.*, 2018). In contrast, there is a lack of data on pregnancy outcomes following modern anticancer therapies, including immunotherapy, ADCs, and TKIs. These treatments may pose unique risks due to their mechanisms of action and pharmacokinetics (Johnson *et al.*, 2022).

Post-treatment pregnancies, especially with new anticancer agents, require careful monitoring due to potential immune system effects. A multidisciplinary approach including oncologists, gynecologists, neonatologists, endocrinologists, and fertility specialists is highly recommended in these cases and can enable patients to realize their desire for a family even after oncological treatments (Massarotti *et al.*, 2019).

## Conclusions

With the increasingly rapid development and availability of new effective anticancer treatments for patients with solid tumors and the major increase in cure rates, there is a growing need to address their potential long-term negative effects, with infertility being one of the major concerns in patients diagnosed at reproductive age (Blondeaux *et al.*, 2021; Khan *et al.*, 2022). However, despite constant calls from the scientific community, very limited data exist on the gonadotoxicity of these new treatments. Recent recommendations by the American Society of Clinical Oncology highlight the urgent need for a structured assessment of ovarian toxicity in clinical trials enrolling post-pubertal, premenopausal patients (Cui *et al.*, 2023). This includes systematic evaluation of both clinical markers and biochemical indicators of ovarian function, with data collection at baseline and at multiple time points during and post-treatment. While integrating such assessments into trial protocols may introduce logistical challenges, the long-term reproductive health of young patients necessitates this effort. Expanding our understanding of the gonadal impact of novel anticancer agents will be key for improving the oncofertility counseling and optimizing decision-making regarding fertility preservation strategies and post-treatment reproductive planning (Cui *et al.*, 2023). Providing new preclinical and clinical evidence on the effects of these therapies on fertility and gonadal function will help to personalize the discussion on the timing and safety of post-treatment pregnancies, ultimately ensuring that reproductive health considerations become an integral part of cancer survivorship care.

## Data Availability

All data reported are publicly available.
